# Docosahexaenoic acid (DHA) alleviates inflammation and damage induced by experimental colitis

**DOI:** 10.1007/s00394-024-03468-x

**Published:** 2024-08-06

**Authors:** Leman Arslan Ariturk, Sumeyye Cilingir, Meltem Kolgazi, Merve Elmas, Serap Arbak, Hande Yapislar

**Affiliations:** 1 Faculty of Medicine, Department of Physiology, Marmara University, Istanbul, Turkey; 2 Faculty of Medicine, Department of Histology&Embriology, Acibadem Mehmet Ali Adinlar University, Istanbul, Turkey; 3 Faculty of Medicine, Department of Physiology, Acibadem Mehmet Ali Adinlar University, Istanbul, Turkey

**Keywords:** Experimental colitis, DHA, IBD, Inflammation, Intestinal barrier

## Abstract

**Purpose:**

Inflammatory bowel diseases (IBD), including Crohn’s disease (CD) and ulcerative colitis (UC), are chronic gastrointestinal disorders associated with significant morbidity and complications. This study investigates the therapeutic potential of docosahexaenoic acid (DHA) in a trinitrobenzene sulfonic acid (TNBS) induced colitis model, focusing on inflammation, oxidative stress, and intestinal membrane permeability.

**Methods:**

Wistar albino rats were divided into Control, Colitis, and Colitis + DHA groups (n = 8–10/group). The Colitis and Colitis + DHA groups received TNBS intrarectally, while the Control group received saline. DHA (600 mg/kg/day) or saline was administered via gavage for six weeks. Macroscopic and microscopic evaluations of colon tissues were conducted. Parameters including occludin and ZO-1 expressions, myeloperoxidase (MPO) activity, malondialdehyde (MDA), glutathione (GSH), total antioxidant status (TAS), total oxidant status (TOS), Interleukin-6 (IL-6), and tumor necrosis factor alpha (TNF-α) levels were measured in colon tissues.

**Results:**

Colitis induction led to significantly higher macroscopic and microscopic damage scores, elevated TOS levels, reduced occludin and ZO-1 intensity, decreased mucosal thickness, and TAS levels compared to the Control group (p < 0.001). DHA administration significantly ameliorated these parameters (p < 0.001). MPO, MDA, TNF-α, and IL-6 levels were elevated in the Colitis group but significantly reduced in the DHA-treated group (p < 0.001 for MPO, MDA; p < 0.05 for TNF-α and IL-6).

**Conclusion:**

DHA demonstrated antioxidant and anti-inflammatory effects by reducing reactive oxygen species production, enhancing TAS capacity, preserving GSH content, decreasing proinflammatory cytokine levels, preventing neutrophil infiltration, reducing shedding in colon epithelium, and improving gland structure and mucosal membrane integrity. DHA also upregulated the expressions of occludin and ZO-1, critical for barrier function. Thus, DHA administration may offer a therapeutic strategy or supplement to mitigate colitis-induced adverse effects.

## Introduction

Inflammatory bowel diseases (IBDs), namely ulcerative colitis (UC) and Crohn’s disease (CD) are chronic inflammations of the gastrointestinal tract with an unknown etiology. IBDs can lead to bleeding and various complications by starting with disruption and inflammation in the mucosal barrier and continuing with an impaired immune response [1]. IBDs have become a rapidly increasing global public health problem since the last century [2]. Although more common in developed countries, their incidence and prevalence are steadily increasing in newly industrialized countries. Furthermore, the incidence and prevalence of IBDs have increased in parallel with the rise in obesity prevalence. It is reported that 15–40% of patients with IBDs are categorized as obese, while 20–40% are overweight [3]. The etiology of IBDs is unknown, but their pathogenesis includes genetic and environmental factors such as climate, pollution, smoking, and the presence of obesity [3, 4]. IBDs lead to complications such as stenosis, fistula, perforation, toxic megacolon, and cancer. The main goals in the treatment of IBDs are to reduce these symptoms and complications and maintain remission. Despite continuous drug use and long-term follow-up, the quality of life of people with IBDs is impaired due to the failure to achieve clinical remission [5].

The exact mechanism of IBDs development is not clear, but it is known that there is an increased production of inflammatory cytokines and reactive oxygen molecules. Cytokines in ulcerative colitis cause an increase in the severity and duration of the disease because of persistent tissue and mucosal damage. In ulcerative colitis, the levels of proinflammatory cytokines, reactive oxygen and nitrogen compounds [6] and the myeloperoxidase (MPO) level, which is an indicator of neutrophil infiltration, are found to be increased [7]. In addition, oxidative stress signals in inflammatory bowel diseases initiate inflammation and trigger the development of IBDs by causing disruption and bacterial invasion in the mucosal barrier of the gastrointestinal tract [8].

Docosahexaenoic acid (DHA) is a long chain, polyunsaturated omega 3 fatty acid (n-3 PUFA). DHA can be used in the body by the enzymatic conversion of alpha-linolenic acid (ALA), an essential fatty acid, or directly obtained from fish oil. ALA in the diet undergoes a series of enzymatic transformations to form eicosapentaenoic acid (EPA) and DHA molecules. Subsequently, EPA and DHA molecules become part of the phospholipid structure of cell membranes and affect the inflammation process. DHA is a protective fatty acid that maintains homeostasis by balancing blood lipids and lipoproteins, increasing antioxidant capacity, and acting as a protective agent against oxidative stress [9].

Excessive reactive oxygen molecules increase membrane permeability, leading to impairment of intestinal mucosal barrier function in IBD [10, 11]. The intestinal epithelial barrier is composed of a single layer of cells whose permeability is maintained by tight junction proteins [12]. Tight junctions are adhesion complexes located between the cells in the epithelium and endothelium that control the passage of ions, water, and molecules. Two of the tight junction proteins are occludin and ZO-1, also known as Zonula occludens. They work together to establish and maintain the structural integrity of tight junctions. Both occludin and ZO-1 have been shown to participate in signaling pathways that regulate tight junction assembly, maintenance, and remodeling. These proteins are not only structural components but also have roles in intracellular signaling cascades that affect tight junction dynamics. Dysregulation of occludin and ZO-1 has been linked to various diseases involving disrupted barrier function, such as inflammatory bowel disease [13]. There are a limited number of studies in the literature indicating the effects of DHA on intestinal barrier functions, most of which are in vitro.

Our aim in this study is to elucidate the possible therapeutic effects and the mechanism of DHA administered intragastrically through its impact on inflammation, oxidative stress, and intestine membrane permeability in a TNBS induced colitis model, which has been shown to reflect human colitis in terms of many features, including histological and immunological changes.

## Materials and methods

### Experimental design and induction of colitis

Female Wistar Albino rats (200–250 g; *n* = 10 per group) were used in the study. Rats were kept under standard humidity (60–70%) and temperature (22 ± 1ºC) conditions with a 12 h light/dark cycle. They were also fed standard pellet chow and had access to water ad libitum during the study. The ethics governing the use and conduct of experiments on animals were strictly observed, and all experimental procedures were approved by the Committee for Animal Research of Acibadem Mehmet Ali Aydinlar University with the approval number “07/05/2018 ACU/HADYEK 2018/21”.

After one week of acclimatization, rats were divided into 3 experimental groups: control (*n* = 8), colitis (*n* = 10) and colitis + DHA (*n* = 10). Rats in the control and colitis groups were given the saline solution; rats in colitis + DHA group were given DHA (Sigma-Aldrich, USA) at a dose of 600 mg/kg body weight per day by oral gavage for 4 weeks. Rats in control group were given the saline with the same protocol to simulate the same treatment stress. The dose of DHA was determined by selecting a non-toxic high dose according to the literature [14]. Chronic colitis was induced as previously described [15]. After an overnight (16 h) fasting, under isoflurane anesthesia, 1 mL of 15 mg/mL TNBS solution dissolved in 40% ethanol was given intrarectally with an 8 cm long cannula to induce colitis. To simulate the same treatment stress, 1 mL of saline was administered intrarectally to the rats in the control group with the same protocol. The weight of the rats was recorded before colitis induction and after three days. On the 4th day, rats were sacrificed by the decapitation; colon tissues were collected and stored at -80^o^C until analysis.

### Evaluation of colitis severity

After euthanasia, the distal 8 cm part of the colon was removed and separated longitudinally, and washed in saline. Then the colon tissues were weighed, and colon weights in grams per 100 g of body weight were used to calculate the relative tissue weight index (RTWI). RTWI was then evaluated according to the following formula [16]:

$$\begin{array}{l}\:Relative\:tissue\:weight\:index\:\left( {RTWI} \right) = \\\frac{{Colon\:weight\:\left( g \right)}}{{Body\:weight\:before\:sacrification}}\:X\:100\end{array}$$

The macroscopic damage scale modified by Wallace et al. [17] was used for macroscopic evaluation (Table 1). Colon damage was evaluated by a blinded, experienced physiologist.

**Table 1:** Macroscopic damage scale

Score	Appearance
0	No damage
1	Focal hyperemia, no ulcers
2	Hyperemia or bowel wall thickening without linear ulceration
3	Ulceration with inflammation at one site
4	Two or more sites of ulceration/inflammation
5	Major sites of damage extending more than 1 cm along the length of colon
6–10	If damage extends more than 2 cm along the length of colon, the score is increased by one for each additional 1 cm

### Histological evaluation

Colon samples collected after decapitation were fixed in 10% neutral buffered formalin. Following fixation, the colon tissues were dehydrated ascending series of ethanol (70%, 90%, 96%, 100%) and cleared with xylene. Clearing and paraffin incubation were performed using an automated tissue processor (Thermo Citadel 2000). The tissues were then embedded in paraffin using an embedding workstation (Thermo-Histostar). Sections with a thickness of 5 μm were taken with a microtome (Thermo Scientific) and transferred to a hot water bath and then placed on slides. Sections were stained with hematoxylin-eosin (H&E) for histological evaluation. Tissue sections taken on a slide for staining were kept in xylene for 30 min. Then, they were kept in a decreasing alcohol series (100%, 90%, 70%) for 5 min each and washed with tap water. The tissue sections, which were stained in hematoxylin for 5 min, were washed with tap water for 5 min and kept in 70% alcohol for 1 min. Staining was completed by immersing the sections in eosin for 3 min. Subsequently tissue sections were processed through a graded series of alcohols (70%, 90%, 100%) and finally cleared with xylene. In the end, the tissue sections were scored semi-quantitatively by an expert blinded histologist under a light microscope (Zeiss LSM 700, Oberkochen, Germany) using a scale ranging from 0 to 3 (0: none, 1: mild, 2: moderate, and 3: severe) for each criterion. The criteria for scoring were surface epithelial damage, the presence of edema, and inflammatory cell infiltration in the mucosa. Additionally, the mucosal thickness of H&E stained sections was measured using ImageJ (1.44 software, National Institutes of Health).

### Immunohistochemistry

Paraffin sections were deparaffinised and rehydrated. Then the sections were washed in PBS, treated with citrate buffer solution (pH 6.0) in a microwave oven and cooled for 15 min. Sections were incubated in blocking solution for 10 min followed by incubation with rabbit anti-occludin primary antibody (Invitrogen, Waltham, MA, USA) at a 1:100 dilution and-ZO-1 antibody (Invitrogen, Waltham, MA, USA) at a 1:100 dilution overnight at 4°C. After primary antibody application, sections were washed in PBS and incubated goat anti-rabbit secondary antibody (Thermo, USA) at a 1:1000 dilution at room temperature. All sections were finally incubated with 4′-6‐ diamidino‐2‐phenylindole (DAPI) at room temperature and analyzed under a confocal microscope (Zeiss LSM 700). The density of occludin and ZO-1 was determined using ImageJ software.

### Oxidative stress parameters

#### TAC and TOC levels

Total antioxidant capacity (TAC) and total oxidant capacity (TOC) were measured in colon tissue using a spectrophotometric method, following the manufacturer’s instructions with the rat TAC and TOC kit (Rel Assay Diagnostics). For TAC measurements, Trolox, a water-soluble derivative of vitamin E, was used as a calibrator, and results were expressed in mmol Trolox equivalents per liter (mmol Trolox equiv/L). For TOC measurements, hydrogen peroxide (H_2_O_2_) was used as the standard, with results expressed in micromoles of H_2_O_2_ equivalents per liter (μmol H_2_O_2_ equiv/L). After obtaining TAC and TOC values, the results were calculated using the following formula [18–20]:

$$\begin{array}{l}\:Oxidative\:stress\:index\:\:\left( {OSI} \right) = \\\frac{{TOC\:\left( {\mu \:mol.H2O2.\:\:Equivalent/L} \right)}}{{TAC\:(\mu \:mol.Trolox.Equivalent/L}}\:X\:100\end{array}$$

#### Glutathione (GSH) levels

The colon tissues were homogenized by adding a 10% trichloroacetic acid solution up to 9 times their weight. Afterward, homogenates were centrifuged (Thermo Fisher Scientific, SL16, Waltham, Massachusetts, USA) at at 3000 rpm for 15 min at 4 °C. The supernatants were taken and centrifuged at 10,000 rpm at 4 °C for a 8 min more. The supernatants were separated and analyzed using the modified Ellman method (40 mg DTNB + 100 ml 1% Na citrate) and read at 412 nm in the spectrophotometer (Shimadzu, UV-2600/UV-VIS/Spectrophotometer, Japan) [21].

### Inflammation markers

#### Myeloperoxidase (MPO) levels

Colon tissues were homogenized by adding nine times their weight in a 5% HETAB solution (in 50 mM potassium phosphate buffer; pH: 6) then centrifuged at 12,000 rpm for 10 min at 4 °C. The pellet was taken and rehomogenized in 50 mM potassium phosphate buffer. Then, 50 mM potassium phosphate buffer, 30 mM o-dianicidine, 60 mM H_2_O_2,_ and sample were added to each tube. The reaction was started in the water bath at 37 °C for 3 min and stopped by adding 2% sodium azide at the end of the 3 min. Samples were centrifuged at 5000 rpm for 15 min at 4 °C. The samples were read at 460 nm in the spectrophotometer (Shimadzu, UV-2600/UV-VIS/Spectrophotometer, Japan) and the results were analyzed [22].

#### Malondialdehyde (MDA) levels

The colon tissues were homogenized by adding nine times its weight of 10% trichloroacetic acid solution. Then the homogenates were centrifuged at 3,000 rpm, + 4 °C for 15 min. The supernatants were taken and centrifuged at 9,000 rpm, + 4 °C, for an additional 8 min. Afterward, 750 μl of thiobarbituric acid and 750 μl tissue samples were added to glass tubes and boiled for 20 min. The color change was read in a spectrophotometer (Hitachi / U-1900) at 535 nm. The results were obtained by multiplying the absorbance values by the coefficient 1.56 × 10^− 5^ M-1 cm^− 1^.

#### Tumor necrosis factor-α (TNF-α) and interleukin-6. (IL-6) levels

Colon tissues were homogenized in buffer solution to determine the levels of cytokines in the colonic mucosa. TNF-α, IL-6 levels were measured using Enzyme-Linked Immunosorbent Assay (ELISA) kits (BT-lab, Zhejiang, China) in accordance with the manufacturer’s instructions. Cytokine concentrations were determined by creating a standard curve. TNF-α levels were measured using BT-lab, E0764Ra, Zhejiang, China; IL-6 levels were measured using BT-lab, E0135Ra, Zhejiang, China ELISA kits.

### Statistical analysis

All data expressed as means ± standard error. Comparison of body weight change data was performed by two-way analysis of variance (ANOVA). Other parameters were compared by one-way ANOVA. Tukey and Games-Howell multiple comparison tests were used to determine the difference between groups. Values of *p* < 0.05 were regarded statistically significant. SPSS 23 version was used for statistical evaluation. Graphs were created using the GraphPad Prism8 Software (GraphPad Prism Software Inc., San Diego, CA).

## Results

### Measurement of the body weight

The body weights of the rats were measured before and three days after the induction of colitis. There was a significant weight loss in the colitis-induced groups (colitis and colitis + DHA group) compared to the control group (*p* < 0.001). Weight loss in the colitis group (8.70%) was slightly higher than the weight loss in the DHA group (7.10%), but the difference was not significant (data not shown).

### Evaluation of the macroscopic damage score and relative tissue weigh index

**Fig. 1:**
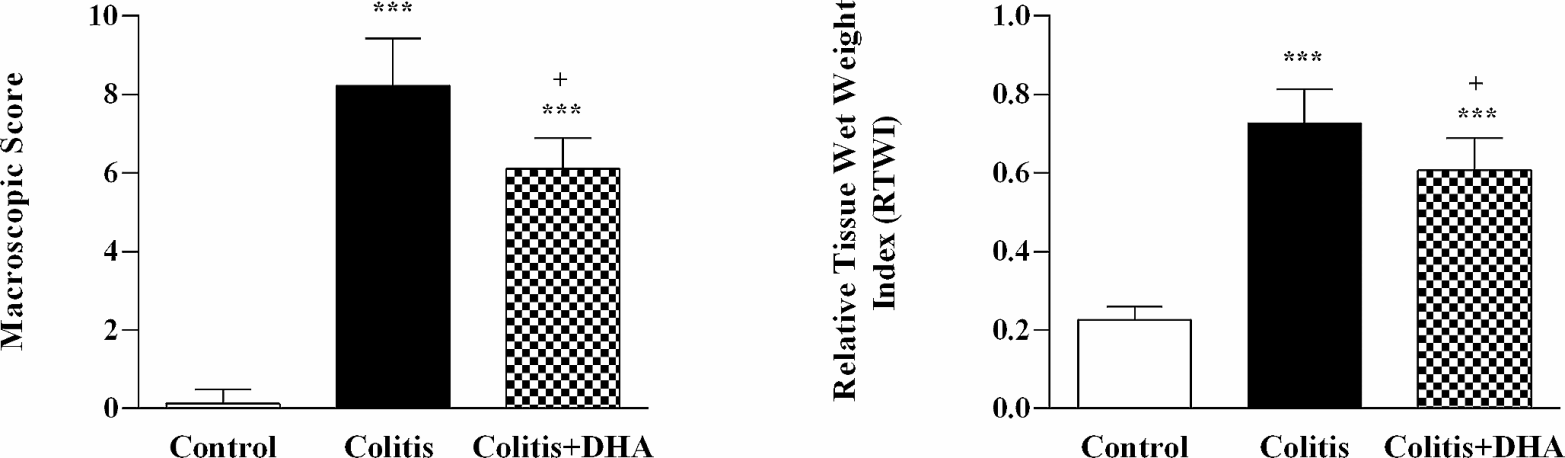
Macroscopic Evaluation (Evaluation of the macroscopic damage score and colon weight after sacrification). (**A**) The macroscopic damage score of experimental groups. **: *p* < 0.001 compared to control group. **+** : *p* < 0.05 compared to colitis group. (**B**) RTWI, Relative tissue weight index. g/100 g body weight. Values are mean ± SD ***: *p* < 0.001 compared to control group

It was observed that the macroscopic damage scores of the colitis and DHA groups were significantly higher than the damage score of the control group (*p* < 0.001). However, it was determined that DHA treatment significantly decreased the damage score compared to the vehicle applied colitis group (*p* < 0.001) (Fig. 1A).

RTWI levels of the colitis and DHA groups were significantly higher than the control group (*p* < 0.001). The RTWI levels of the DHA group were slightly lower than the colitis group, the difference was statistically significant (*p* < 0.05). (Fig. 1B).

### Histologic evaluation

**Fig. 2:**
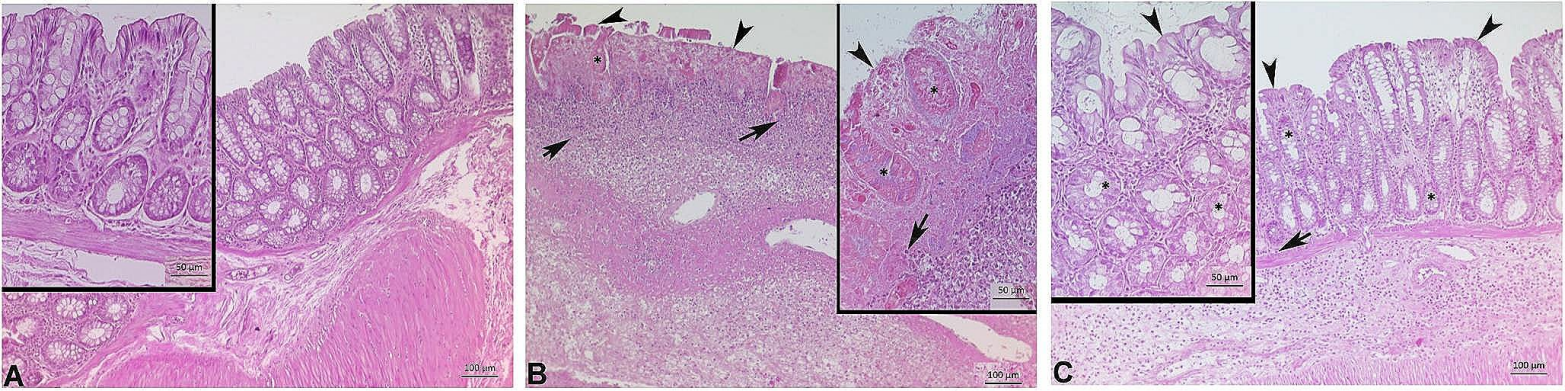
Representative photomicrographs of colon tissues (**A**) Normal colon tissue morphology was seen in control group. (**B**) Epithelial desquamation (arrowhead), disrupted glands (asterisk), and leukocyte infiltration (arrow) were observed in colitis group. (**C**) Mild degree of mucosal damage with decreased number of leukocytic infiltration (arrow), glandular damage (asterisk) and epithelial damage (arrowhead) were observed in Colitis + DHA group. H&E staining

Colon tissue samples from the experimental groups underwent histopathological evaluation under a light microscope. Normal tissue morphology was observed in the control group (Fig. 2A). Desquamation of the surface epithelium, degeneration of the glandular structure, and neutrophilic infiltration in the mucosa were detected in the colitis group (Fig. 2B). Mild desquamation of the surface epithelium restored glandular morphology and limited areas of neutrophilic infiltrations were noticed in the colitis + DHA group (Fig. 2C).

**Fig. 3:**
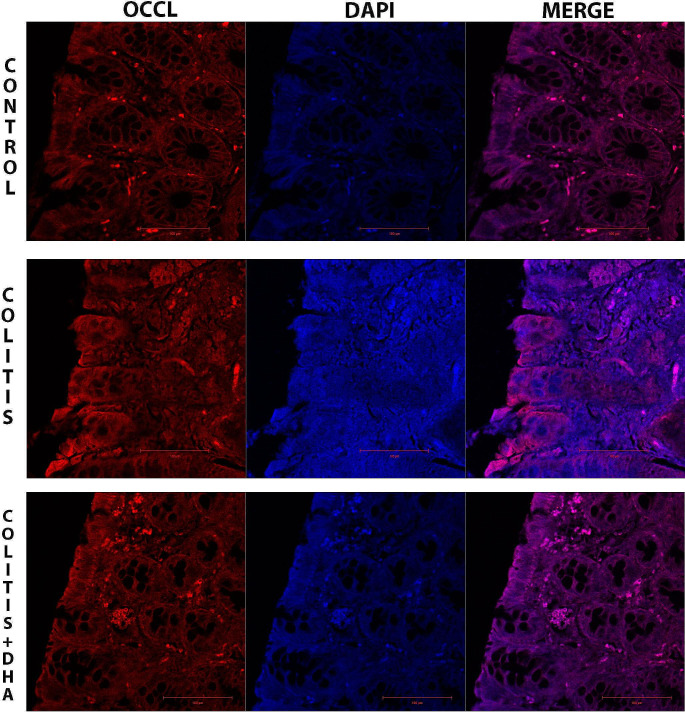
Representative micrographs of Occludin immunoflourescence staining in the experimental groups. Occludin immunopositivity and DAPI are seen in bright red and blue colours, respectively. All sections were counterstained with 4-6-diamidino-2-phenylindole DAPI to visualize the nucleus

**Fig. 4:**
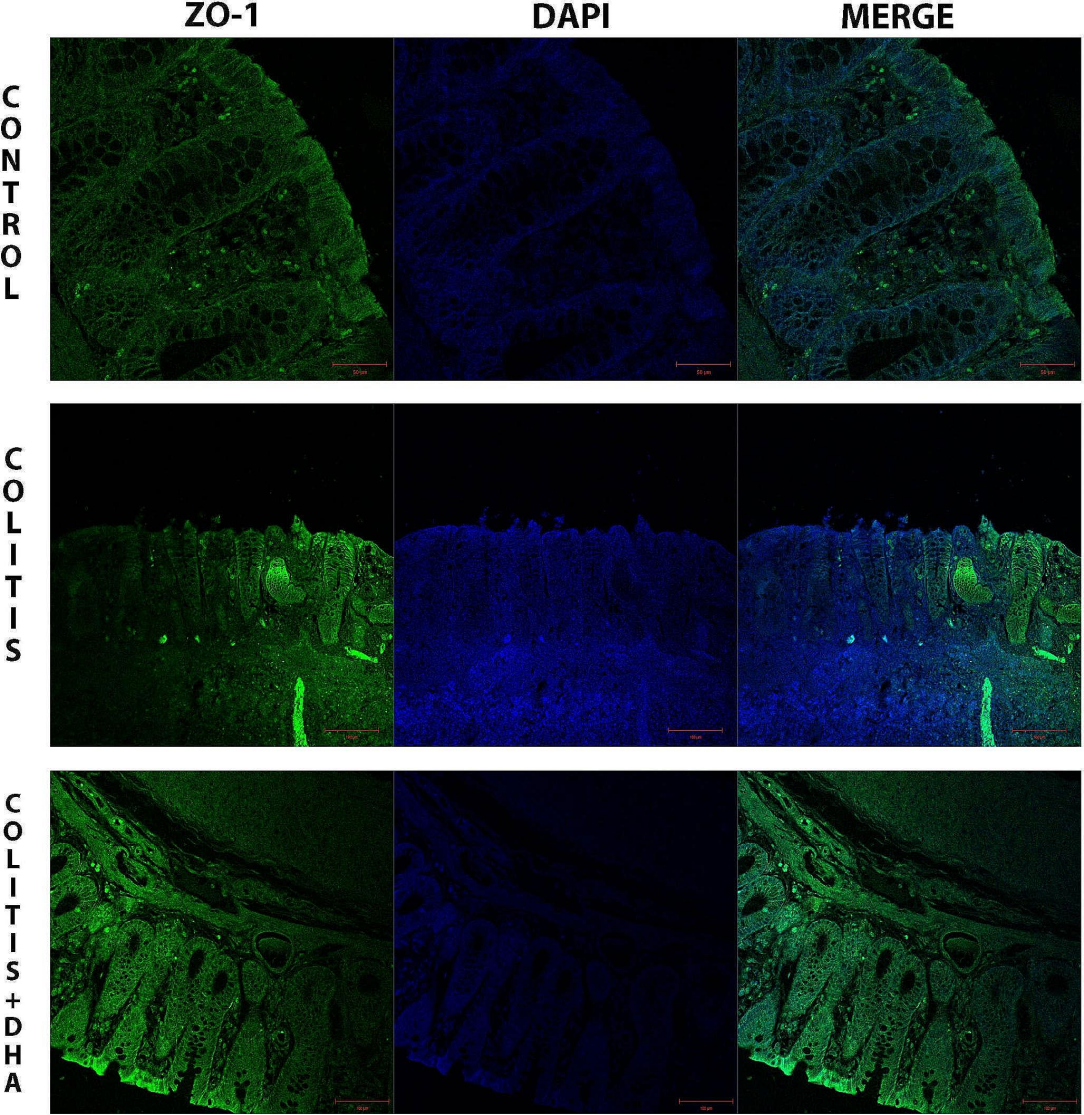
Representative micrographs of ZO-1 immunoflourescence staining in the experimental groups ZO-1 immunopositivity and DAPI are seen in bright green and blue colours, respectively. All sections were counterstained with 4-6-diamidino-2-phenylindole DAPI to visualize the nucleus

The immunofluorescent pictures of the colon sections were examined under the confocal microscope to evaluate the epithelial integrity of the colon tissue. Occludin and ZO-1 immunopositivity were observed in the epithelial regions of the colon tissues. In the colitis group, decreased occludin and ZO-1 immunopositivity was observed. However, occludin and ZO-1 immunopositivities were higher in the colitis + DHA group than in the colitis group (Figs. 3 and 4).

**Fig. 5:**
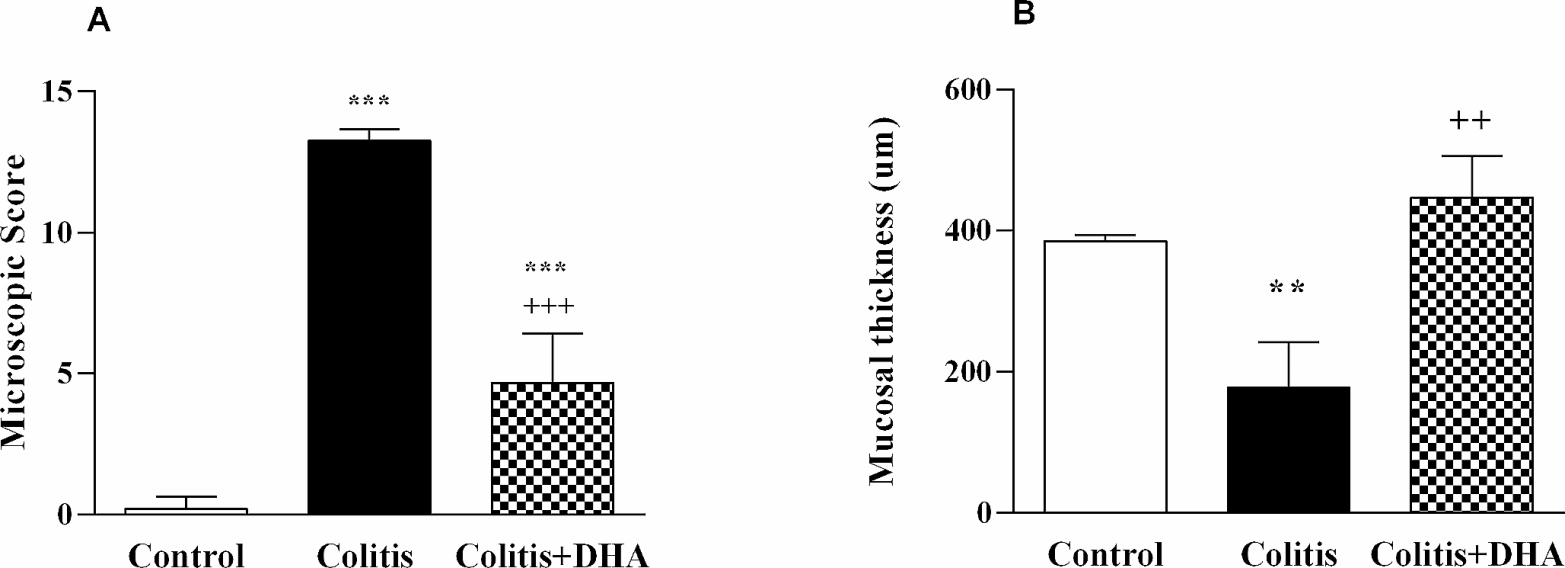
(**A**) The microscopic damage scores of the experimental groups. ***: *p* < 0.001 compared to control group. +++ : *p* < 0.001 compared to colitis group. (**B**) Mucosal thickness of colon tissue in experimental groups. **: *p* < 0.01 compared to control group. ++: *p* < 0.01 compared to colitis group

Based on histopathological scoring of experimental groups, microscopic damage score was found to be significantly decreased in colitis group when compared to the control group (*p* < 0.001). The DHA group had a lower microscopic damage score compared to the colitis group (*p* < 0.001). (Fig. 5A). The mucosal thickness of the intestine was decreased in the colitis group compared to the control group (*p* < 0.01). DHA administration in colitis increased the mucosal thickness significantly compared to the colitis group (*p* < 0.01) (Fig. 5B).

**Fig. 6:**
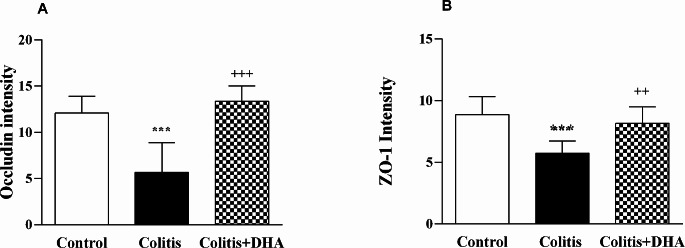
Fluorescence intensity of occludin and ZO-1 expressions ***: *p* < 0.001 compared to control group. +++ : *p* < 0.001 compared to colitis group. ++ : *p* < 0.01 compared to colitis group

Occludin and ZO-1 immunoreactivity was calculated in the epithelial colon tissue of the experimental groups. While occludin reactivity was significantly decreased in the colitis group compared to the control group (*p* < 0.001), it was significantly higher in the DHA group than in the colitis group (*p* < 0.001) (Fig. 6A). ZO-1 immunoreactivity was significantly decreased in the colitis group compared to the control group (*p* < 0.001). In the DHA-administered group, ZO-1 immunoreactivity was significantly increased compared to the colitis group (*p* < 0.005) (Fig. 6B).

### Inflammatory parameters

**Fig. 7:**
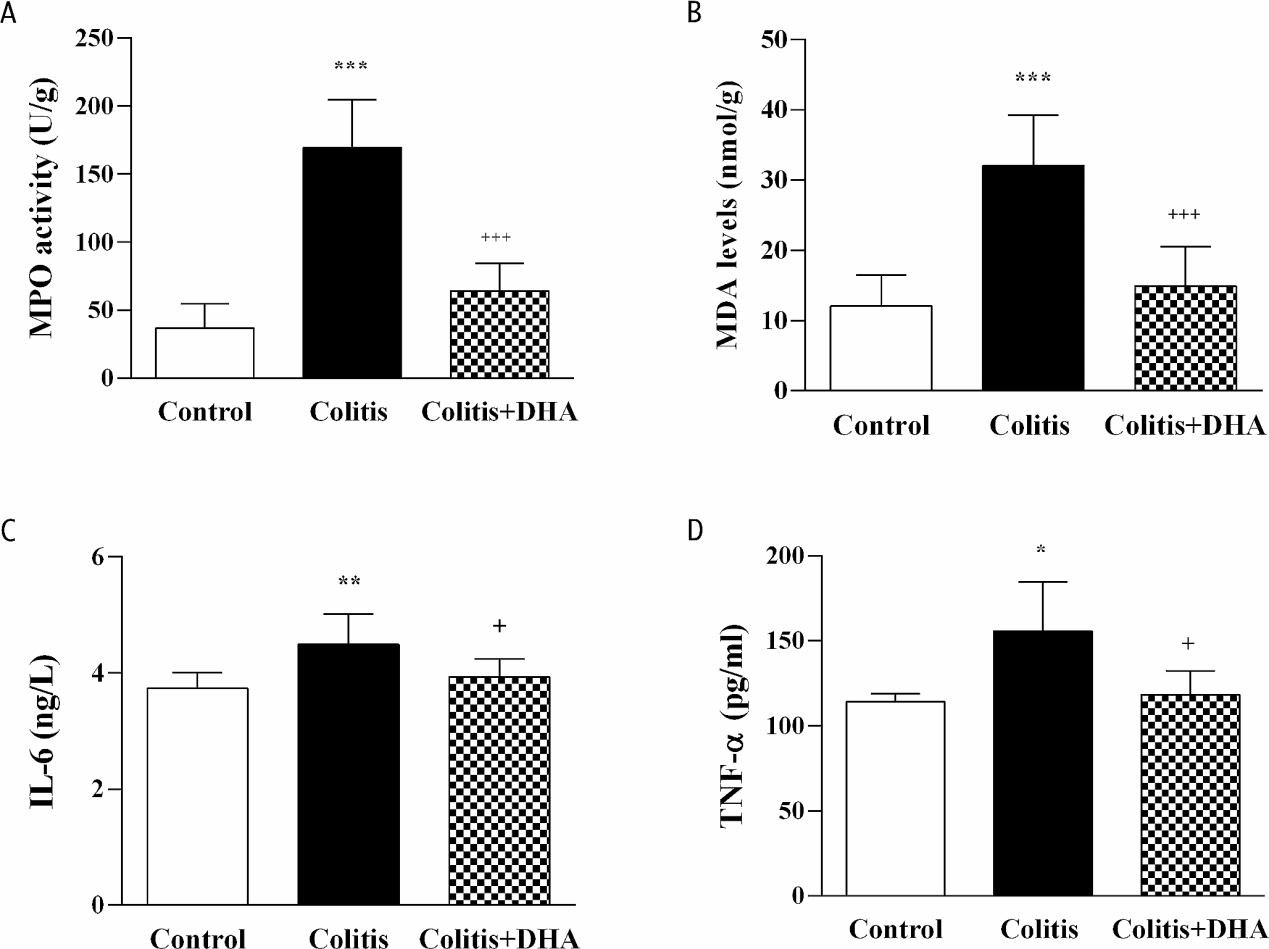
Inflammatory Markers. The Malondialdehyde (MDA), Myeloperoxidase (MPO), Interleukin-6 (IL-6), Tumor necrosis factor alpha (TNF-α) levels of the experimental groups. Values are mean ± SD. *: *p* < 0.05 compared to control group **: *p* < 0.01 compared to control group. ***: *p* < 0.001 compared to control group. +: *p* < 0.05 compared to colitis group. ***: *p* < 0.001 compared to colitis group

MPO levels, indicating neutrophil infiltration into the colon tissue, were measured. In the colitis group, MPO levels were significantly higher than the control group (*p* < 0.001), and DHA administration significantly decreased the MPO level compared to the colitis group (*p* < 0.001) (Fig. 7A).

MDA levels, an indicator of oxidant damage, was measured in the colon tissue. In the colitis group, MDA levels were elevated compared to the control group (*p* < 0.001). On the other hand, DHA treatment significantly decreased MDA levels which were increased with colitis induction (*p* < 0.001) (Fig. 7B).

IL-6 and TNF-α levels were significantly increased in the colitis group compared to the control group (*p* < 0.01 and *p* < 0.05, respectively). In the colitis + DHA group, both cytokine levels were significantly lower than in the colitis group (*p* < 0.05) (Fig. 7C and D).

### Oxidative stress parameters; colonic TAC, TOC, OSI and GSH levels

The total oxidant and antioxidant capacities of the colon tissue samples were assessed. TAC levels of the colitis group were significantly low, and the TOC and OSI levels increased significantly compared to the control group (*p* < 0.05). On the other hand, DHA treatment significantly ameliorated TAC and TOC levels (*p* < 0.05) and slightly decreased OSI levels. However, the difference in OSI was not statistically significant (Table 2).

**Table 2:** Oxidative stress parameter. Total antioxidant capacity (TAC), total oxidant capacity (TOC), oxidative stress index (OSI) and glutathione (GSH) levels of the experimental groups. Values are mean ± SD

Groups	TAC (mmol/L)	TOC (μmol/L)	OSI (μmol/L)	GSH (μmol/g)
Control (*n* = 8)	1,13 ± 0,12	11,51 ± 3,48	0,86 ± 0,51	1,47 ± 0,46
Colitis (*n* = 10)	0,97 ± 0,19**	15,47 ± 2,75**	1,64 ± 0,53*	0,49 ± 0,22**
Colitis + DHA(*n* = 10)	1,23 ± 0,28^#^	12,66 ± 4,21^#^	1,41 ± 1,17	0,98 ± 0,27**^#^

*: *p* ≤ 0.05 compared to control group. **: *p* ≤ 0.01 compared to control group. #: *p* ≤ 0.05 compared to colitis group

GSH levels, an indicator of the amount of endogenous antioxidant glutathione, were measured in colon tissue. The GSH levels in the colitis group were reduced compared to the control group, while DHA administration significantly preserved the GSH level (Table 2).

## Discussion

IBD is a disease characterized by increased production and release of inflammatory cytokines and reactive oxygen species. The simultaneous decrease in tissue antioxidant levels is a common feature in IBDs, along with the increasing production of free radicals [23].

In the study, the therapeutic effects of DHA on epithelial barrier integrity, oxidative stress, and inflammatory process were investigated in the experimental colitis model in rats. The macroscopic and microscopic damage scores significantly increased with colitis induction, and DHA treatment reduced these scores. The histologic evaluation also demonstrated that DHA application reduces shedding in the surface epithelium and improves gland morphology. It was also observed that while mucosal thickness significantly decreased with colitis induction, DHA treatment improved intestinal barrier function.

Although increased oxidative stress levels are known in inflammatory bowel diseases, the mechanism of their relationship has not yet been fully elucidated. Many factors, such as colonic epithelium, microvascular endothelium, or inflammatory cells (especially neutrophils), are responsible for this increment [24, 25]. On the other hand, the increase in production of the ROS such as superoxide radicals and hydrogen peroxide is effective in the formation of the inflammatory response. Kruidenier et al. argued that exposure to ROS molecules is responsible for mucosal tissue damage in IBDs [26]. Furthermore, Han et al. demonstrated that superoxide dismutase treatment may have a therapeutic effect on the TNBS-induced colitis model [27].

The effect of DHA on cellular oxidative stress parameters has been studied extensively in the literature. DHA suppresses ROS production, increases the activity of antioxidant enzymes, and improves antioxidant capacity in different disease conditions [28–30]. In our study, related to TAC and TOC levels, the data we obtained in this context overlap with the data of our study results related to oxidative stress. TAC and TOC level measurements showed that while the TOC levels of the colitis group were higher, the TAC levels were significantly lower than those of the control group. Administration of DHA to colitis-induced rats reversed this situation. It was observed that there was a significant decrease in TOC levels and an increase in TAC levels compared to the colitis group.

GSH is one of the essential endogen antioxidant molecules that protect the cell against oxidative stress. It is present in the cytoplasm, nucleus, and mitochondria of the cells. GSH levels are known to decrease during inflammation [31]. It has been demonstrated that severe degeneration of the colonic mucosa, development of diarrhea, and weight loss occur in mice with chemically induced GSH deficiency [32]. Decreased GSH levels are accepted as indicators of both the severity of inflammation and the level of oxidative stress [33]. The results showed that the GSH levels in the colon tissue of the rats with colitis were significantly lower than those in the control group. GSH levels of the DHA administered group significantly increased compared with the colitis group (Table 2).

MDA is the end product of the oxidation of PUFAs by free radicals in the body [34]. MDA level serves as an indicator of lipid peroxidation caused by free radicals in the cell membrane [35]. In our study, TNBS application significantly increased MDA levels in the colitis group compared to the control group, and DHA treatment significantly decreased MDA levels in the colon tissue. This result suggests that DHA may be associated with reducing the ROS induced tissue lipid peroxidation in the the mucosa [36–38]. Fatty acids are the main components of the cell membrane phospholipids. By incorporating into the phospholipid structure of the cell membrane, DHA may have a significant effect in maintaining the structural integrity of the cell. It increases the durability of the membrane, decreases the cell sensitivity to free radicals, and lipid peroxidation.

Many studies have suggested that inflammatory cells such as neutrophils and macrophages have considerable effects on the pathogenesis of IBDs [39]. The myeloperoxidase enzyme, synthesized in neutrophils with potent antibacterial and antiviral effects, is accepted as the indicator of neutrophil infiltration in colitis [40]. Increased mucosal MPO levels in UC patients have been reported [41]. It has also been suggested that neutrophil infiltration is the most important factor responsible for the increase in ROS production [24, 25].

In our study, MPO enzyme activity was significantly increased in the colitis-induced group, and DHA application decreased MPO enzyme activity to the levels observed in the control group. In some studies it has been shown that DHA administration reduces neutrophil infiltration in the various experimental inflammatory disease models [42, 43]. In accordance with these studies, DHA may exert its MPO-reducing effect by reducing neutrophil infiltration. It was reported that 100 mg/kg/day DHA treatment improved gastric damage caused by indomethacin in rats, reduced MPO, MDA, and TNF-α levels and protected the endogenous GSH levels [44]. DHA administered 500 mg/kg/day by gavage for 14 days improved lung damage caused by paraquat, decreased MPO activity and MDA level and preserved GSH level in mice [45].

A wide variety of cytokines such as TNF-α and IL-6 increase in circulation or tissue during inflammation. Usually, inflammatory cell migration and cytokine chemotaxis cause progressive tissue destruction in IBD [46]. To investigate the effect of DHA on the colitis-induced inflammatory process, pro-inflammatory cytokine TNF-α was measured in the study. There was an increase in TNF-α levels in the colitis group compared to the control group, but this increase was not statistically significant. On the other hand, DHA administration significantly decreased TNF-α levels compared to the colitis group (*p* < 0.05; Fig. 7D). Reduction of TNF levels by anti-TNF drugs is one of the treatment options in IBD, and TNF inhibitors are commonly used for the treatment of IBD [47, 48].

IL-6 provides communication between innate and adaptive immune response by driving the differentiation of CD4 T cells. In several previous studies, it was determined that while spontaneous colitis occurred in IL10^−^/^−^ mice, the levels of IL-6 were also increased [49–51]. In contrast to previous studies, the IL-6 level was significantly higher in the colitis group compared to the control group in our study. DHA administration significantly decreased the IL-6 level compared to the colitis group (Fig. 7C).

Fatty acids are the main component of cell membrane phospholipids [52]. LA and ALA are essential fatty acids that cannot be synthesized in the body. Arachidonic acid (AA), EPA, and DHA molecules formed by the enzymatic transformation of these essential fatty acids. These molecules participate in the membrane phospholipid structure and affect the inflammatory process. AA and EPA molecules have both inflammatory and anti-inflammatory effects, while the DHA molecule has potent anti-inflammatory effects [53]. In addition, previous studies have shown that DHA inhibits nuclear factor kappa B (NF-kB), which is the main transcription factor that regulates the expression of some inflammatory mediators (such as cyclo-oxygenase-2, tumor necrosis factor-a, IL-1b, IL-6) [54]. It is known that inhibiting NF-kB has a protective effect on inflammation by preventing the production of the the proinflammatory mediators. Similarly, Qiu et al. [55] determined that maresin 1 demonstrated protective effects by inhibiting proinflammatory mediators by inhibiting NF-kB.

Paracellular transition in the epithelium is regulated by tight junction proteins. The barrier function of the epithelium can be easily disrupted, leading to increased permeability in IBD [56]. Fractures in the epithelium and rearrangement of tight junction proteins, which control paracellular transition, along with glandular atrophy, have been demonstrated in both UC and CD [12, 57, 58]. Claudins are transmembrane proteins that are a fundamental component of tight junctions. Proper claudin polymerization and arrangement are essential for maintaining the tightness of the barrier [59]. Marin et al. [60] proved the disorganization of tight junction proteins in the inflamed regions of the intestine in a study involving CD patients. It was reported that the expression of claudin 5, 8, and 3, which contribute to epithelial tightness, decreased in active CD. Occludin and ZO-1 are key players in maintaining the integrity of the intestinal barrier, which is crucial for preventing the leakage of harmful substances from the gut into the bloodstream, thereby maintaining the selective permeability of these barriers [61]. ZO-1 acts as a scaffold protein that connects the cytoplasmic tails of claudins, occludin, and other tight junction proteins to the actin cytoskeleton inside cells. This connection helps anchor these transmembrane proteins at the tight junctions and provides stability to the junctional complex [62].

Kuacharzik et al. [63] demonstrated a decrease in occludin protein levels during both the active and remission periods of UC, whereas it decreased only during the active phase of CD. In an in vitro study, prophylactic treatment with EPA and DHA in epithelial cell culture reduced the permeability induced by TNF and IFN and prevented the deterioration of tight junction morphology [64]. Zhao et al. [65] showed that the destruction of tight junction proteins occludin and ZO-1 was improved when 35.5 mg/kg DHA was administered for two weeks in a mouse model of colitis. Consistent with the literature, we found that intragastric administration of DHA protects the integrity of the epithelial barrier by reducing the destruction of occludin and ZO-1 proteins, thereby showing protective effects against TNBS-induced colitis. ZO-1 and occludin work in concert to establish and maintain the structural integrity of tight junctions, and the absence or dysfunction of either of these proteins can have profound consequences. Their interactions and functions are interdependent and crucial for the proper functioning of these barriers. The loss of both ZO-1 and occludin can severely disrupt the formation and function of tight junctions, leading to significant impairment of barrier integrity and function [66].

**Fig. 8:**
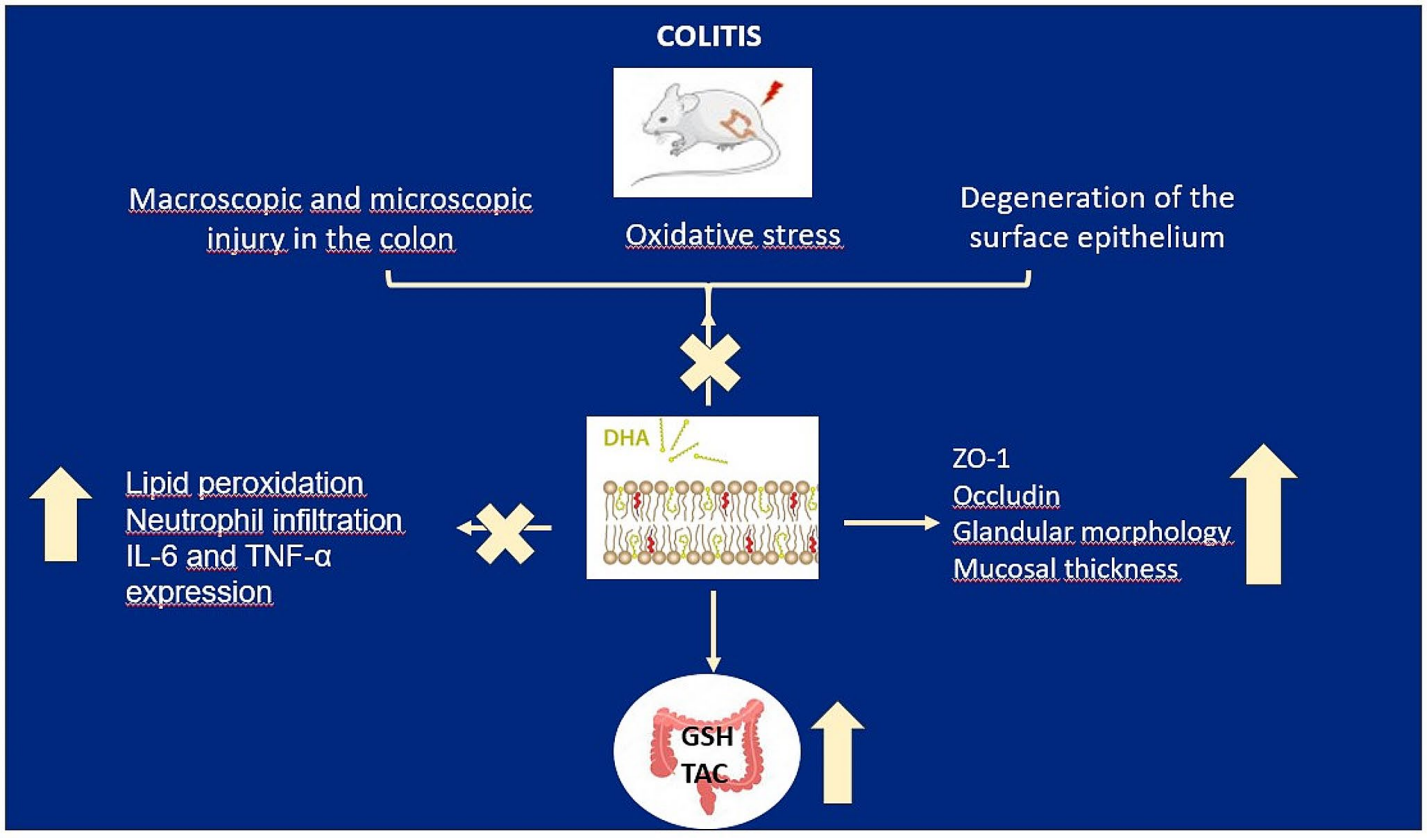
Details of the mechanism behind the effect of DHA in preventing colitis. DHA, with its long and highly unsaturated structure, embeds itself among the phospholipids in cell membranes, contributing to the fluidization and increased flexibility of the membrane and triggers the production of protective compounds that have anti-inflammatory effects. DHA supports the structural integrity of the cell membrane, providing protection against harmful oxidative processes. By reducing the peroxidation of membrane lipids, it enhances the cell’s resistance to aging and damage

In conclusion, DHA demonstrated its effects by preventing neutrophil infiltration, suppressing neutrophil-derived ROS production, preserving GSH content, reducing the levels of proinflammatory cytokines IL-6 and TNF-α, and mitigating shedding in the colon epithelium while improving gland structure and mucosal membrane integrity. Additionally, DHA administration increased the expression levels of occludin and ZO-1, which are crucial for preventing increased intestinal permeability (Fig. 8). Therefore, DHA administration could be considered a beneficial supplement to prevent the adverse effects of colitis induced by the inflammatory process.
